# Temporal trends and gender differentials in causes of childhood deaths at Ballabgarh, India - Need for revisiting child survival strategies

**DOI:** 10.1186/1471-2458-12-555

**Published:** 2012-07-26

**Authors:** Anand Krishnan, Nawi Ng, Suresh K Kapoor, Chandrakant S Pandav, Peter Byass

**Affiliations:** 1 Centre for Community Medicine, All India Institute of Medical Sciences, New Delhi, 110029, India; 2 Umea Center for Global Health Research, Department of Public Health and Clinical Medicine Umea University, Umea, Sweden; 3 Department of Community Health, St. Stephen’s Hospital, Delhi, India

**Keywords:** Child survival, Neonatal mortality, Child mortality, Cause of death, Gender, India

## Abstract

**Background:**

Relating Information on causes of deaths to implementation of health interventions provides vital information for program planning and evaluation. This paper from Ballabgarh Health and Demographic Surveillance System (HDSS) site in north India looks at temporal trends and gender differentials in the causes of death among under-five children.

**Methods:**

Data on causes of death for 1972-74, 1982-84, 1992-94, 2002-04 were taken from existing HDSS publications and database. Physicians’ assigned causes of death were based on narratives by lay health worker till 1994 and later by verbal autopsy. Cause Specific Mortality Fractions (CSMF) and Cause Specific Mortality Rates (CSMR) per 1000 live births were calculated for neonatal (<1 month) and childhood (1-59 months) period. Gender difference was estimated by calculating ratio of CSMR between girls and boys. Available information on coverage of childhood interventions in the HDSS was retrieved and compiled.

**Results:**

The CSMF of prematurity and sepsis was 32% and 17.6% during neonatal period in 2002-04. The share of infections in all childhood deaths decreased from 55.2% in 1972-74 to 43.6% in 2002-04. All major causes of mortality (malnutrition, diarrhea and acute lower respiratory infection) except injuries showed a steep decline among children and seem to have plateued in last decade. Most of disease specific public health interventions were launched in mid eighties. . Girls reported significantly higher mortality rates for prematurity (RR 1.52; 95% CI 1.01-2.29); diarrhea (2.29; 1.59 – 3.29), and malnutrition (3.37; 2.05 – 5.53).

**Conclusions:**

The findings of the study point out to the need to move away from disease-specific to a comprehensive approach and to address gender inequity in child survival through socio-behavioural approaches.

## Background

Child mortality serves as a measure of a nation's health and development, state of health services as well as social and economic situation. In 2010, it was estimated that 7.6 million children die each year worldwide before reaching their fifth birthday, a decrease of approximately 2 million during the past decade [1]. Globally, approximately 64% of deaths among children aged <5 years are attributable to infectious diseases, most notably diarrhea, pneumonia, malaria, and Acquired Immuno Deficiency Syndrome (AIDS) and 40.3% of all childhood deaths occur in neonates [1,2]. The vast majority of gains in child survival have been accomplished through scale-up of interventions such as immunization, micronutrient supplementation, access to safe water, insecticide-treated bednets, oral rehydration therapy, antibiotics, antimalarial therapy, and antiretroviral therapies [2-4]. These can be largely said to be part of the selective primary health care interventions promoted by Unicef [5]. This approach has been criticized as deviating from the original comprehensive approach envisaged in Alma Ata [6,7].

In India during 1990–2007, the Under-five Mortality Rate (U5MR) had declined by 43.5% (from 124 to 70 per 1,000 live births); with the infant and neonatal deaths declining 31.2% (from 80 to 55 per 1,000 live births), and 32.1% (from 53 to 36 per 1,000 live births), respectively. An analysis of the Annual Rate of Reduction (ARR) of mortality suggests that during 2000–2003 there was a rapid ARR in all child mortality rates, most prominently in Early Neonatal Mortality Rates (ENMR) with an ARR of 7.7%. However, the ARR slowed for all age groups during the next 4 years (2004–2007) and the ENMR may have actually increased during this time period [8].

This slowdown could be due to the fact that current interventions have achieved their maximum potential effectiveness or that the decline has not been well spread across the population. While traditionally, inequity has been studied in the context of socio-economic differentials, increasingly gender inequities are being reported in the world and most notably from South Asia [9-11]. A nationwide study from India reported that all-cause mortality rate in children aged 1–59 months was about 36% higher in girls than in boys (56·7 and 41.7 deaths per 1,000 live birth, respectively) and most of the leading causes of death were between 12% and 72% higher in girls than in boys, with the exception of injuries and meningitis/encephalitis [12].

The stagnation in childhood mortality rates and the existence of gender inequity in mortality calls for a relook at strategies for childhood mortality reduction in India and other developing countries. Temporal relationship between the level of implementation of major child health interventions and changes in causes of childhood deaths can provide valuable insight for program planning and evaluation. However, good quality and representative data on causes of death over a period of time are not available from developing countries including India. Only in recent times has India generated information on causes of death data on a nationally representative basis through the prospective Million Death Study [12]. Health and Demographic Surveillance System (HDSS) sites like Ballabgarh that have populations under regular demographic surveillance can provide this information. This paper uses available information on causes of death among under-five children of Ballabgarh to measure the decadal changes in major causes of childhood deaths among the population of Ballabgarh project from 1972 to 2004 and the gender differences in the causes of death in the last decade.

## Methods

### Study area

The study site is in Ballabgarh, a block within the Faridabad District, located in the state of Haryana in northern India. This population is a part of Comprehensive Rural Health Services Project (CRHSP), a collaborative effort between All India Institute of Medical Sciences (AIIMS) in New Delhi and the state of Haryana. The CRHSP includes a 50-bed hospital, 2 primary health centers, and 12 sub-centers and covered a total population of 78,562 in 2004. Multi-Purpose Workers (MPW) make home visits to facilitate delivery of primary and preventive care. Each person in the catchment area is recorded in the CRHSP computerized database and has a unique health information system number [13]. While the population has been under surveillance since 1961, the population data was fully computerized only since 1992.

### Method of ascertaining causes of death

Till 1995, MPWs identified death and informed health assistant who was trained to fill the death card with description of events at the time of death (narrative). Finally a physician assigned the cause of death [14]. Since 1995, a new verbal autopsy tool was introduced but the system of data collection and assignment of cause of death remained same. The current verbal autopsy tool has retained the narrative component but added questions/checklist of symptoms for disease-specific modules and has been validated [15,16]. Results based on these verbal autopsy forms have been published earlier [17].

### Study period & source of information

The individual data on causes of death before 1992 are not present in database. This study therefore uses this information from earlier publications for the years 1972-74 and 1982-84 and in 1992-94 [17-19]. This paper adds the data for the year 2002-04 from HDSS database to cover four time periods ten years apart. Information on initiation and coverage of specific program interventions in the study area such as immunization were collected through the past annual reports of the project and previous peer-reviewed publications [20-30]. When information on interventions was not available, we used estimates based on the discussion among people who were present in Ballabgarh during those periods including one of the authors (SKK) who has been present at Ballabgarh for the entire period covered in the paper i.e. 1972-2004.

Only secondary data are used in this paper. The data were collected as a part of routine demographic surveillance of the population. The research protocol was cleared by the Institute Ethics Committee of AIIMS.

### Statistical issues

Standard definitions of neonatal (<1 month) and childhood (1-59 months) mortalities were used and a denominator of 1000 live births (lbs) was used to calculate cause and age-specific death rates, which included Neonatal Mortality Rate (NMR), and Child Mortality Rate (CMR). Cause Specific Mortality Fractions (CSMF) were calculated for each major group of causes of death as proportion of the total deaths caused by the specific cause in that age group. The Cause Specific Mortality Rates (CSMR) per 1000 lb were calculated by dividing the number of deaths attributed to a particular cause to total live births. The time trend of CSMR was tested for significance using chi-square for trend. For the sex differentials in causes of death, the period was extended to include all deaths in 2002-07 so as increase the sample size. The gender differential was assessed by calculating rate ratio (RR) with 95% Confidence Interval (CI) using Statcalc function of EpiInfo. This was estimated as a ratio of the CSMR among girls to same CSMR among boys in the same age group.

## Results

During the study period of thirty three years from 1972 to 2004, the population under surveillance doubled from 42,112 to 78,562. There was a major decline in fertility and mortality rates during this period (Table 1). The steepest absolute decline in birth rate occurred during 1974 -1982 (9 points) followed by 1994 to 2002 (7 points). Neonatal mortality declined by 58% and childhood mortality by 38% from 1972 to 2004. Major decline in NMR occurred in the period 1982 to 1994 (66%) whereas for childhood mortality it was in decade of seventies (62%). While the pattern in the first two decadal periods was consistent with declining fertility and mortality rates, the last decadal period (1994-04) showed a sharper decline in birth rate, a sharp rise in NMR and a plateauing of CMR.

**Table 1:** Fertility and mortality indicators* of Ballabgarh HDSS during 1972-2004

**Indicators**	**1972-1974**	**1982-1984**	**1992-1994**	**2002-2004**
Total population (year)	42112 (1973)	53203 (1983)	65813 (1993)	78562 (2003)
Number of live births (CBR)	5626 (44.5)	5688 (35.6)	6532 (33.1)	6184 (26.2)
Neonatal deaths (<1 months; NMR)	214 (38.0)	154 (27.1)	60 (9.2)	137 (22.2)
Childhood deaths (1-59 months; CMR)	731 (129.9)	451 (79.4)	345 (52.8)	304 (49.1)
Total under-five deaths (U5MR)	945 (167.9)	605 (106.5)	405 (62.0)	441 (71.3)

*All the rates were measured as number of deaths per 1000 live-births over the three year period.

Note: CBR = crude birth rate; NMR = neonatal mortality rate; CMR = child mortality rate; U5MR = under 5 mortality rate.

**Figure 1:**
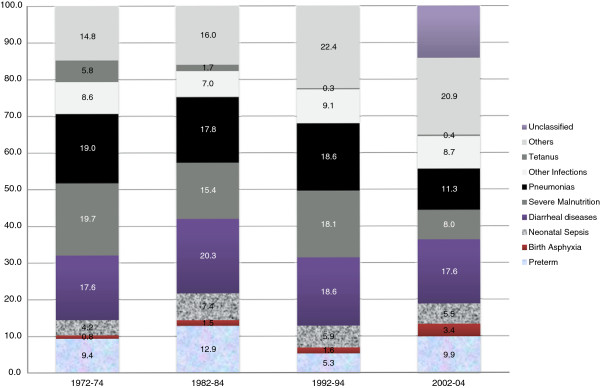
Changes in Cause Specific Mortality Fraction among under-five children in Ballabgarh, 1972-2004.

The CSMF showed that the share of infections in all childhood deaths decreased from 55.2% in 1972-74 to 43.6% in 2002-04 (Figure 1). The CSMF of diarrhea was more or less constant during this period (17.6% to 20.3%), but it declined sharply for tetanus. Unclassified causes of death were first reported in 2002-04, once the new VA tool was introduced.

**Figure 2:**
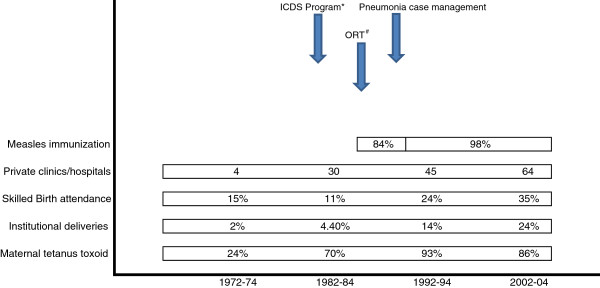
Time table of child survival interventions and their coverage in study area. * ICDS - Integrated Child Develpoment Services providing growth monitoring and food supplementation to under-six children. # Promotion of Oral Rehydration Therapy.

Among neonates, deaths due to tetanus showed the steepest decline and have been eliminated for the last two decades (<1 /1000 lb) (Table 2). The CSMR of birth asphyxia varied from 0.9 to 2.4 per 1000 lb during the study period (p value for trend = 0.052). Despite halving in their cause-specific mortality rates, prematurity and infections continued to be the main causes of death in the neonatal period with a CSMF of 32% and 17.6% respectively in 2002-04. Coverage of maternal tetanus immunization showed a significant jump in the first decade and by 1992-94 a near universal coverage was achieved and maintained (Figure 2). Institutional deliveries and those attended by skilled birth attendant were less than 5% till 1984 after which they increased to about quarter of all deliveries by the end of the study period.

**Table 2:** Trends in cause-specific mortality rates (per 1000 lb) of major diseases in neonatal age group (1972-2004) at Ballabgarh HDSS

	**1972-74**	**1982-84**	**1992-1994**	**2002-2004**	**% decline during 1972 and 2004**
Preterm or low birth weight	15.8	13.7	3.1	7.1	55*
Birth asphyxia	1.4	1.6	0.92	2.4	-71
Infections (Septicemia + ARI + diarrhea)	7.1	7.9	3.4	3.9	45*
Tetanus	9.8	1.8	0.15	0.32	97*
Others including congenital anomalies	3.9	2.1	1.5	3.4	13
Unclassifiable	--	--	--	5.0	---

*Trend significant at 0.05 level.

Among children in the 1-59 months age group, diarrhea and acute lower respiratory infections (ALRI or pneumonias) were the commonest causes of death (Table 3). Infections constituted 58.3% of all 1-59 aged deaths in 1972 and 54.8% in 2002 despite a 2.5 times reduction in absolute mortality rate. All major causes of mortality (malnutrition, diarrhea and Acute Lower Respiratory Infection/ALRI) except injuries showed a significant steep decline in the in this age group (Table 3). Malnutrition and pneumonias decreased by three fourths during this time whereas diarrhea and other infections decreased by half. Significant mortality decline due to ALRI (in seventies) preceded diarrhea (in eighties) even though most of the related interventions like measles immunization, ALRI case management and oral rehydration therapy were started only in mid eighties. The access to health care was very limited in the first decade with it being almost restricted to government health facilities, which have largely remained unchanged during the study period. In Ballabgarh town in 1972, apart from the project hospital there was only one qualified private care provider and this had increased substantially to about 34 qualified medical doctors (in modern medicine) and 30 registered medical practitioners (qualified in Indian or other complementary systems of medicine but often practicing modern medicine).

**Table 3:** Trends in cause specific mortality rates (per 1000 lb) among children (1-59 months) (1972-2004) at Ballabgarh HDSS

**Time period**	**1972-74**	**1982-84**	**1992-1994**	**2002-2004**	**% decline during 1972 and 2004**
Acute Lower Respiratory Infections (pneumonia)	31.8	19.0	10.8	8.1	75*
Diarrhea	29.5	21.6	10.8	12.6	57*
Severe Malnutrition	33.0	16.4	10.5	5.7	83*
Injuries	1.95	2.3	2.9	3.3	-69
Other infections	14.5	7.5	5.3	6.2	57*
Others including Unclassified	19.0	12.7	8.6	13.4	29

*Trend significant at 0.05 level.

Sex differentials in causes of death were studied for the period 2002-07. Girls showed significantly higher mortality rates for prematurity (1.52; 1.01-2.29); diarrhea (2.29; 1.59–3.29) malnutrition (3.37; 2.05–5.53), and other causes of death 1.48 (1.04–2.10). The ill-defined deaths were also more common in girls compared to boys with a RR of 1.87 (1.27–2.77) as evident from Table 4. While other infections were also higher among girls, the observed difference was not statistically significant.

**Table 4:** Sex differentials in cause-specific mortality rate (per 1000 lb) among children below five years during 2002-07

**Causes of death**	**M (N = 6,604)**			**F (N = 5,509)**			**Risk Ratio of female/male (95% C.I.)**
	**N**	**CSMR**	**%**	**N**	**CSMR**	**%**	
Preterm	41	6.2	12.2	52	9.4	10.9	1.52 (1.01 – 2.29)
Septicemia	12	1.8	3.6	19	3.5	4.0	1.90 (0.92 – 3.91)
Birth Asphyxia	22	3.3	6.6	21	3.8	4.4	1.14 (0.63 – 2.08)
Acute lower respiratory Infections (Pneumonia)	53	8.0	15.8	54	9.8	11.4	1.22 (0.84 – 1.78)
Diarrhea	44	6.7	13.1	84	15.3	17.7	2.29 (1.59 – 3.29)
Severe Malnutrition	21	3.2	6.3	59	10.7	12.4	3.37 (2.05 – 5.53)
Injuries	12	1.8	3.6	13	2.4	2.7	1.30 (0.59 – 2.84)
Other Infections	33	5.0	9.9	40	7.3	8.4	1.45 (0.92 – 2.30)
Other Causes	56	8.5	16.7	69	12.5	14.5	1.48 (1.04 – 2.10)
Unclassified	42	6.2	12.2	64	11.6	13.5	1.87 (1.27 – 2.77)
**All Causes**	335	50.7	100	475	86.2	100	1.67 (1.46 – 1.52)

## Discussion

This paper looks at changes in causes of death among under-fives in a population experiencing major decline in fertility and mortality rates over thirty-three years period. Stagnant childhood mortality rates, however, call for a careful review of current strategies of disease specific or selective primary health care interventions largely focusing on better management of diseases implemented through private sector health services.

Logically, differences in causes of death between different time periods and populations can be due to three main reasons: difference in incidence, difference in case fatality rates and misclassification. Misclassification in cause of death has been reported to be low for congenital anomalies and injuries and high for neonatal sepsis, diarrhea, pneumonias and malnutrition [16,31]. Incidence of a disease would be affected by differences in its determinants (for example, source and quality of water for diarrhea) or presence of preventive measures (vaccination for tetanus). The case fatality rate is determined by a combination of three factors: health seeking behavior, availability and access to health services and quality of care provided in the health services. We attempt to explain the differences noted in this study between time periods and sex using this framework.

### Temporal trends in causes of death

The decrease in neonatal mortality was mainly because of decrease in prematurity and infections related deaths including tetanus. Despite an increase in institutional deliveries to a quarter of all births, deaths due to birth asphyxia did not come down. Less than half of these deliveries are in government health facilities. Probably, these institutional deliveries are occurring in low risk mothers in higher socio-economic groups. Also, it is possible that the facilities where the deliveries are now occurring are not fully equipped in terms of trained human resource and equipment resulting in higher cases of birth asphyxia. As neonatal asphyxia has major developmental consequences to the surviving child it would result in serious burden on the society to care for the affected neonates with various forms of disability. Misclassification of neonatal deaths as still births or prematurity as sepsis in the initial period which could have gradually decreased over a period of time due to better training and monitoring is a possibility. The increase in coverage of pregnant mothers with tetanus toxoid from about 24% in 1972 to almost universal coverage in early nineties and a strategy of supply of disposable delivery kits (a sterilized kit having a gauze piece, razor blade and thread) resulted in the decline in neonatal tetanus mortality [21].

The initial decline followed by a rise in prematurity related deaths is difficult to explain. There is no information on incidence of low birth weight for the period before 1990 in the study area. Since then, different sources have reported the incidence of low birth weight ranging from 9% to 26%, including data from electronic database (19% for 2010) [29,30]. The decrease in deaths due to low birth weight is thus, unlikely to be due to decreased incidence but perhaps due to decreased case fatality due to improved access. Despite an apparent and not well-documented increase in health facilities where deliveries can be conducted, health facilities at Ballabgarh still lack capacity for managing babies below 1.5 Kg. These babies would be referred to centres in Faridabad and Delhi which are now more accessible due to better roads and affordable due to improvement in socio-economic status. However, seeking costly care also has the potential to accentuate gender differences.

The main causes of death in 1-59 months age group were ARI, diarrhea and malnutrition. It has been reported from the study area that measles vaccination introduced in 1985 resulted in 57% decline in all cause mortality and that case management approach resulted in 26% decline in ARI mortality [17,21]. These relate well with the results reported. A case fatality rate of 1.3% was reported for pneumonia from Ballabgarh in 1987, a figure which is on the lower side of interquartile range (1.3-2.6%) of case fatality rate globally [32]. This was because mothers were able to recognize danger signs for pneumonia and take them for treatment with private practitioners [25,26]. It is likely that the case fatality rate has further declined in Ballabgarh since then. The reported ARI incidence from the study area has remained around 3 per child per year from 1987 to 2005 [33]. For the same period, the pneumonia incidence was reported as 0.3 per child per year [26]. There is a wide difference in the estimated incidence of pneumonia between developing and developed countries (0.29 Vs. 0.05 per child per year) [33]. The large potential in reduction of pneumonia incidence and an already low case fatality rate point to the need for a change of strategy to address reduction in incidence of respiratory infections. Among the established risk factors for pneumonia , the important one in the study area are those of indoor air pollution due to cooking fuel and tobacco smoking, poor housing and child care practices.

The promotion of Oral Rehydration Therapy (ORT) has been the major intervention for reduction of diarrhea-related deaths. High use of fluid therapy was reported in late eighties [22]. The same study reported a case fatality rate of 0.6%, an estimate confirmed by another study done in the area around these villages [34]. As diarrheal deaths have further reduced, case fatality rates must have come down further. Though exact information is not available, it is unlikely that the incidence rate of 2.6 diarrheal episodes per child per year reported in this population in late eighties has come down significantly [22]. Three fifths of the population did not have latrines at home and 81% had access to drinking water at or close to home as per our data in 2002-3 [27]. The stagnation in diarrheal incidence rates and a decline in diarrhea mortality rates have been noted globally as well [35]. This means that the focus of the efforts to control diarrhea has to shift to water supply, sanitation and hygiene (WASH) interventions and possibly vaccination against common agents of diarrhea. These have already been identified as potentially cost-effective interventions [35].

Data from studies done in this area over the study period have reported more or less similar rates of grade II or more malnutrition in late sixties and eighties to a prevalence of 24.9% in 2004 [36,37]. Anecdotally, it appears that there is a decline in rates of severe malnutrition, with grade I and II malnutrition remaining at about the same levels. One would therefore expect that the contribution of malnutrition to deaths would decrease. Malnutrition as a cause of death also has high potential for misclassification [16,31]. Many of those who died due to ARI and diarrhea would also have severe malnutrition and could have been differently classified by different medical officers.

The Million Death Study (MDS), done in 2001-03 reported that three leading causes (prematurity and low birthweight, neonatal infections and birth asphyxia) accounted for 78% of all neonatal deaths as compared to 62% in this study in 2002-04 [12]. Two causes accounted for 50% of all deaths at 1–59 months: pneumonia and diarrhoeal diseases as compared to 42% in Ballabgarh during roughly the same time period. The mortality rate due to prematurity, infections and asphyxia as estimated by MDS were however, in absolute terms, much higher than in Ballabgarh. The overall mortality rate was also higher with u5MR being 85.8 per 1000 lb and NMR being 36.9 per 1000 lb as compared to 71 and 22, respectively in our study. However, deaths due to congenital abnormalities in the neonatal age group (1.2 vs. 2.1) and injuries in the 1-59 months age group (2.9 vs. 2.3) were reasonably similar to our study. Tetanus deaths were still higher at 1.2 per 1000 lb in MDS. This similarity in major causes of death supports external validity of our study in Ballabgarh.

### Trends in childhood mortality reported from other parts of the world

The global estimates between 2000 to 2010 show a decrease in all causes of under-five mortality during this decade, mainly due to the decline in diarrhea and pneumonia deaths, with annual decline rates of 4% and 3.1%, respectively [1] There are not many studies which have reported on changing patterns of causes of childhood deaths over time. In Matlab HDSS, using routinely collected demographic surveillance data from a rural area of Bangladesh between 1975 and 2002, the overall reduction in early and late neonatal mortality comparing the same period was 39% and 73%, respectively. The dramatic decline in neonatal mortality was mainly due to a fall in deaths from neonatal tetanus [38]. Data from three Bangladesh Demographic and Health Surveys done during 1993 to 2004 show an increase in death rates due to birth asphyxia (from 4 to 9 per 1000 lb) and prematurity (from 4 to 7 per 1000 lb). The decline in mortality in the 1-59 months age group were also similar to those reported in this study [39]. A population-based survey conducted through a nationwide multi-level surveillance network in China showed that the greatest rate reduction in under-five mortality in rural China between 1996 and 2006 was seen in deaths due to pneumonia and diarrhoea; 69.4% and 69.1%, respectively. Deaths due to premature birth/ low birth weight and birth asphyxia decreased by 46.8% and 46.0%, respectively, while deaths due to congenital heart disease increased by 24.5% [40]. Deaths due to injuries have been reported to have increased in the last decade in both Africas and Americas and probably reflect increased mechanization of agricultural as well as introduction of motorised transport in villages [1].

### Gender differences in childhood causes of death

In societies in which care is equal for boys and girls, baby girls have a lower mortality rate than baby boys: the ratio of neonatal mortality for boys to girls is usually at least 1.2 [41]. In the current study however, this was reversed. Data from different sources in India show that the major causes of death in the first week of life are due to asphyxia and prematurity whereas most of deaths in the 7-28 days are due to sepsis [42,43]. A review of all child deaths in the study area between 1991-95 showed that for early neonatal deaths (<7 days), there was a slight preponderance of boys (55: 45) whereas for late neonatal deaths, the ratio was reversed to 40:60, which was more or less maintained till 5 years of age [44]. The gender difference in late neonatal mortality has also been reported from Pakistan which shares a similar cultural practice of female neglect [45]. Gender difference in care seeking for neonatal illnesses was confirmed in a recent study in Ballabgarh which showed that sick male neonates were more likely to be taken to a health facility especially to a private one, which is perceived by community to be better than public ones [46]. Higher unclassified deaths among girls could be due to their care givers not providing sufficient information on their deaths, either due to disinterest or because of hesitation that this may reflect on their neglect.

A community based study done in eighties in our study area, reported similar incidence rates for boys and girls for diarrhea and pneumonia but a higher case fatality rate among girls [22,26]. This has been confirmed by large scale national level household surveys in India [47,48]. Data from our primary health centre (PHCs) for the year 1983 showed that emergency consultation among under-fives, which were mainly for ALRI and diarrhoea, were higher for boys as compared to girls [49]. A review of all admissions due to ALRI to the secondary level hospital at Ballabgarh for the year 1990 showed that 74% of admissions were of boys [50]. Other studies have also shown that girls are often brought to a health facility in more advanced stage of illness than boys, are taken to less qualified doctors when they are ill, and less money is spent on medicines for them than for boys [10,11]. Data from a HDSS site in Bangladesh also showed higher mortality rates among girls for malnutrition (2.5 times) and diarrhea (2.1) as in this study area [51].

The strengths of this study are that the information on causes of death is available over a period of time along with information on coverage with many childhood interventions for the same population. The main weakness is that the method of ascertainment of causes of death underwent changes especially for the last time period. However, a comparison done in 1995 showed acceptable agreement between causes of death arrived by VA tool and the previous system [15]. Inter-observer variation between physicians who coded the cause of death is possible. However, the fact that injuries and birth asphyxia remained stable during this period with no major interventions targeting these illnesses having been implemented in the study area and decline in tetanus deaths in conformity with interventions reflects the robustness of the data. Lack of data on quality and outcome of care of private health care facilities makes it difficult to comment on their role in reduction of childhood mortality in the study area.

## Conclusion

This study has highlighted some critical issues in terms of the persistent inequalities in neonatal and child mortality by gender in a part of India. However, evidence support that this problem is not restricted to the study area but affects large part of the country especially in the northern part as well as neighboring countries in South Asia. The decline in childhood mortality till now has been driven by reduction in case fatality through improved management practices and has been largely implemented by private sector (immunization being the only exception). This unregulated growth of private sector has resulted in increased access but has largely not addressed “quality of care” issues and one can argue that in fact this has resulted in poorer care. Provision of health care mainly through private sector has also worsened the differential health care seeking by child’s gender. Development of Integrated Management of Childhood Illnesses (IMCI) signals a move from disease specific intervention to multiple diseases [52]. But it still looks at reducing mortality and is disease oriented. In a recent review by Rohde et al comparing the success with primary health care approach in 30 developing countries, they emphasize the need to progress from selective primary health care to comprehensive primary health care [6]. The results of this paper emphasize the same call for a revision of strategy to a broader development paradigm (resulting in reduction of incidence) and universal coverage (addressing equity), both of which are principles enshrined in primary health care approach. This will require interventions beyond health sector as well as scaling up of health systems to deliver universal coverage. This demands major financial investments which governments and donor agencies need to deliberate.

### What is already known?

There has been a slowing in decline in child mortality rates in India, despite major gains in the past decades. The gains have been attributed to implementation of child survival strategies like oral rehydration therapy, pneumonia case management, immunization.

### What this study adds?

The gains made by selective disease-specific primary health interventions and expansion of private health care infrastructure have reached their upper limit of effectiveness. Infections and prematurity continue to be the main killers of children below five-years in India. This is compounded by worsening gender inequities with higher death rates in girls being reported even in late neonatal period. This calls for a revision of child survival strategy to a broader development paradigm (resulting in reduction of incidence) and universal coverage (decreasing gender differential). This will require interventions beyond health sector.

## Competing interests

The authors declare that they have no competing interests.

## Authors’ contributions

AK conceived the study, collated the information, analyzed and interpreted the data, wrote the first draft. SKK reviewed the data, provided interpretation and reviewed the manuscript. CSP provided critical inputs for data interpretation and presentation and reviewed the manuscript. NN rechecked the analysis and reviewed the manuscript. PB helped with data interpretation , reviewed the manuscript and is the guarantor of the paper. All authors have approved the manuscript.

## Funding statement

This research received no specific grant from any funding agency in the public, commercial or not-for-profit sectors.

## Pre-publication history

The pre-publication history for this paper can be accessed here:

http://www.biomedcentral.com/1471-2458/12/555/prepub
